# Human endothelial cells and fibroblasts express and produce the coagulation proteins necessary for thrombin generation

**DOI:** 10.1038/s41598-021-01360-w

**Published:** 2021-11-08

**Authors:** Clay T. Cohen, Nancy A. Turner, Joel L. Moake

**Affiliations:** 1 Department of Pediatrics, Section of Hematology-Oncology, Baylor College of Medicine, Texas Children’s Hospital, Houston, TX USA; 2 Department of Bioengineering, Rice University, Houston, TX USA

**Keywords:** Proteins, RNA, Cell biology, Thrombosis

## Abstract

In a previous study, we reported that human endothelial cells (ECs) express and produce their own coagulation factors (F) that can activate cell surface FX without the additions of external proteins or phospholipids. We now describe experiments that detail the expression and production in ECs and fibroblasts of the clotting proteins necessary for formation of active prothrombinase (FV–FX) complexes to produce thrombin on EC and fibroblast surfaces. EC and fibroblast thrombin generation was identified by measuring: thrombin activity; thrombin–antithrombin complexes; and the prothrombin fragment 1.2 (PF1.2), which is produced by the prothrombinase cleavage of prothrombin (FII) to thrombin. In ECs, the prothrombinase complex uses surface-attached FV and γ-carboxyl-glutamate residues of FX and FII to attach to EC surfaces. FV is also on fibroblast surfaces; however, lower fibroblast expression of the gene for γ-glutamyl carboxylase (*GGCX*) results in production of vitamin K-dependent coagulation proteins (FII and FX) with reduced surface binding. This is evident by the minimal surface binding of PF1.2, following FII activation, of fibroblasts compared to ECs. We conclude that human ECs and fibroblasts both generate thrombin without exogenous addition of coagulation proteins or phospholipids. The two cell types assemble distinct forms of prothrombinase to generate thrombin.

## Introduction

Human endothelial cells (ECs) produce factor (F)VIII that is stored in EC Weibel–Palade bodies and secreted along with ultra-large von Willebrand factor multimers^1–5^. We recently reported that several types of human ECs also produce the coagulation proteins required for both extrinsic and intrinsic coagulation pathway-mediated FX activation. Specifically, tissue factor (TF), FVII, FIX, FX, and prothrombin (FII) were detected in ECs; and FX can be activated on human umbilical vein EC (HUVEC) surfaces without the addition of exogenous proteins, proteolytic enzymes, or phospholipids^6^. Furthermore, TF, FVII, FIX, and FX were detected in human fibroblasts, that are, along with ECs, components of human vascular walls. The cellular source of FV has previously been enigmatic. Earlier studies demonstrated that ECs express and synthesize FV, which then binds to EC surfaces^7–9^, and participates in FII activation on the surface of bovine aorta ECs and HUVECs. In these experiments, FII activation was induced by the exogenous addition of activated FX, but the addition of FV was not necessary^10–16^. In this report, we demonstrate that FV is expressed and produced by ECs and fibroblasts.

In this article, we report experiments, also performed without the exogenous additions of proteins or phospholipids, demonstrating that active prothrombinase (FX–FV–FII) complexes can form, and produce thrombin, on human ECs and fibroblasts.

The proteolytic cleavage of FII by the membrane-bound prothrombinase complex in the presence of calcium, generates thrombin plus the prothrombin fragment 1.2 (PF1.2)^17–20^. Thrombomodulin and antithrombin (AT) are primary regulators of thrombin on EC surfaces. Thrombin in thrombin–thrombomodulin complexes activates protein C (PC)^21–23^, whereas heparan sulfate-bound AT attaches to, and neutralizes, thrombin^24–27^ by forming thrombin–antithrombin complexes (TAT). Elevated plasma levels of PF1.2 and TAT are markers of coagulation activation in pro-thrombotic conditions^28–33^.

In this study, our analysis of thrombin activity, the FII cleavage product (PF1.2), and TAT, indicate that human ECs and fibroblasts participate actively in hemostasis by promoting thrombin generation, albeit via different processes.

## Results

In order to investigate the formation of prothrombinase complexes on both EC (human umbilical vein endothelial cells [HUVECs]; glomerular microvascular endothelial cells [GMVECs]; liver sinusoidal microvascular endothelial cells [LSECs]) and fibroblast surfaces, we determined whether or not these cells express and produced FV and FII. The cellular site of FV production has been controversial^34,35^. In our previous study we found that both ECs and fibroblasts produced TF, FVII, FIX, and FX; however, FII was only detected in ECs^6^.

### Gene expression levels of *F5* in ECs and fibroblasts

**Figure 1:**
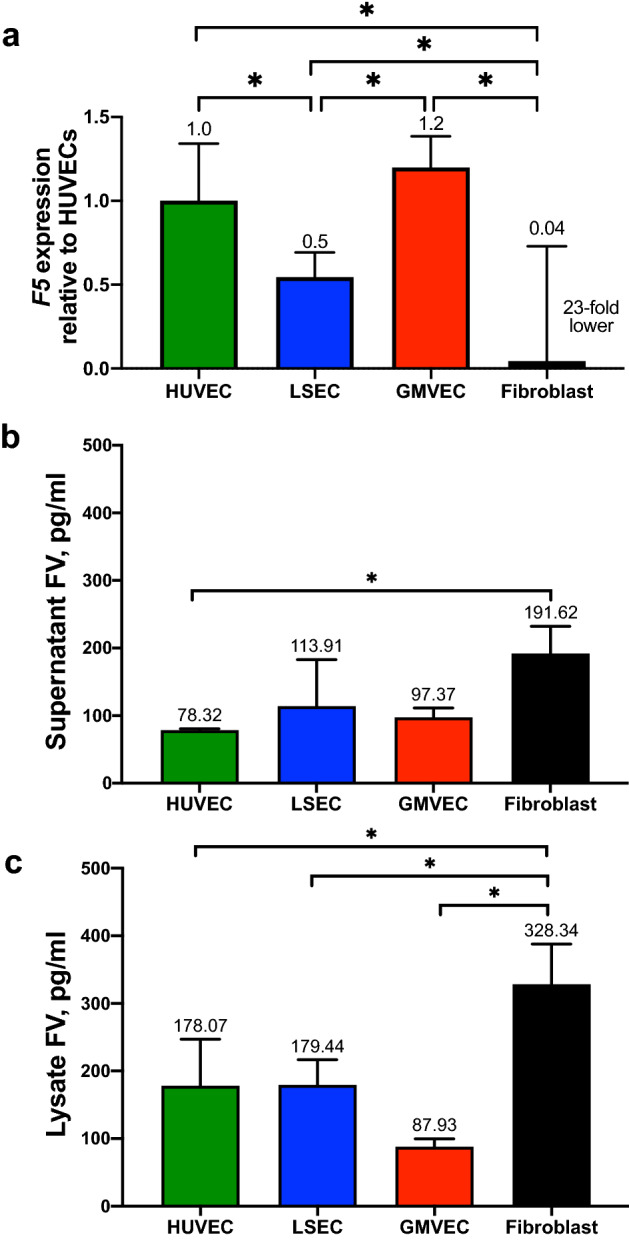
Gene expression levels of *F5* and FV protein levels in the supernatant and lysate/cell membrane fractions of ECs and fibroblasts. (**a**) The expression levels of *F5* in each cell type was quantified relative to *F5* levels in HUVECs after normalization to *GAPDH* in each real-time PCR analysis. Data shown are means + SD from four to six separate RNA extractions for each cell type (HUVEC, n = 5, LSEC, n = 4, GMVEC, n = 6, fibroblast, n = 4). Quantification of FV in cell (**b**) supernatant and (**c**) lysate/cell membranes by ELISA in HUVECs, LSECs, GMVECs, and fibroblasts (n = 3 for each cell type). FV protein concentrations (means + SD) in pg/ml were normalized to total lysate protein to account for cell number differences. *p < 0.05.

Expression levels of *F5* (FV gene) were measured from reverse transcribed RNA isolated from HUVECs, LSECs, GMVECs, and fibroblasts. Figure 1a shows the *F5* mRNA levels of each cell type relative to the *F5* levels in HUVECs. The fibroblast *F5* mRNA levels were 13- to 28-fold lower than *F5* levels in each EC type (Fig. 1a, fibroblasts and HUVECs *p* = 0.0001; fibroblasts and GMVECs *p* < 0.0001; fibroblasts and LSECs *p* = 0.023). HUVECs and GMVECs had twofold higher *F5* mRNA levels compared to LSECs (LSECs and HUVECs *p* = 0.028; LSECs and GMVECs *p* = 0.0033). The complete comparison statistics for HUVEC, LSEC, GMVEC, and fibroblast *F5* expression is shown in Supplementary Table S1.

### Quantification of FV in EC and fibroblast supernatant and lysate/cell membrane fractions

Factor V protein was measured in supernatant (Fig. 1b) and lysate/cell membrane fractions (Fig. 1c) of HUVECs, LSECs, GMVECs, and fibroblasts. In contrast to previous studies by others which concluded that human ECs contain FV, but do not release the protein into cellular media^36,37^, FV could be quantified in our experiments in both supernatant and cell lysates (containing cell membranes) of both fibroblasts and ECs. Fibroblast supernatant contained 2.5-fold higher levels of FV than HUVECs (*p* = 0.04, Fig. 1b), whereas fibroblast lysates had ~ two- to fourfold higher concentrations of FV than HUVECs (*p* = 0.025), LSECs (*p* = 0.027), and GMVECs (*p* = 0.0015, Fig. 1c). Immunofluorescent images show the increased presence of FV on fibroblast surfaces compared to FV on EC surfaces in Supplementary Figure S1.

The FV protein levels in cell supernatants and lysates, as well as the other coagulation protein measurements in this study, were normalized to total cell protein levels. The total average protein (mg/ml ± standard deviation [SD]) in each EC type was: HUVECs 5.8 ± 1.8; GMVECs 4.6 ± 0.5; LSECs 4.2 ± 0.3; and 10.4 ± 4.3 in fibroblasts.

To summarize, human ECs (HUVECs, LSECs, and GMVECs) and fibroblasts express *F5* mRNA and produce FV protein. The majority of FV is found in the lysate/cell membrane fractions of both cell types. Despite fibroblasts having lower *F5* expression levels than ECs, concentrations of FV in fibroblast lysate/cell membrane fractions were two- to fourfold higher than in EC lysate/cell membrane fractions. Fibroblast surfaces have increased FV compared to ECs by immunofluorescence imaging.

### EC and fibroblast *F2* expression

**Figure 2:**
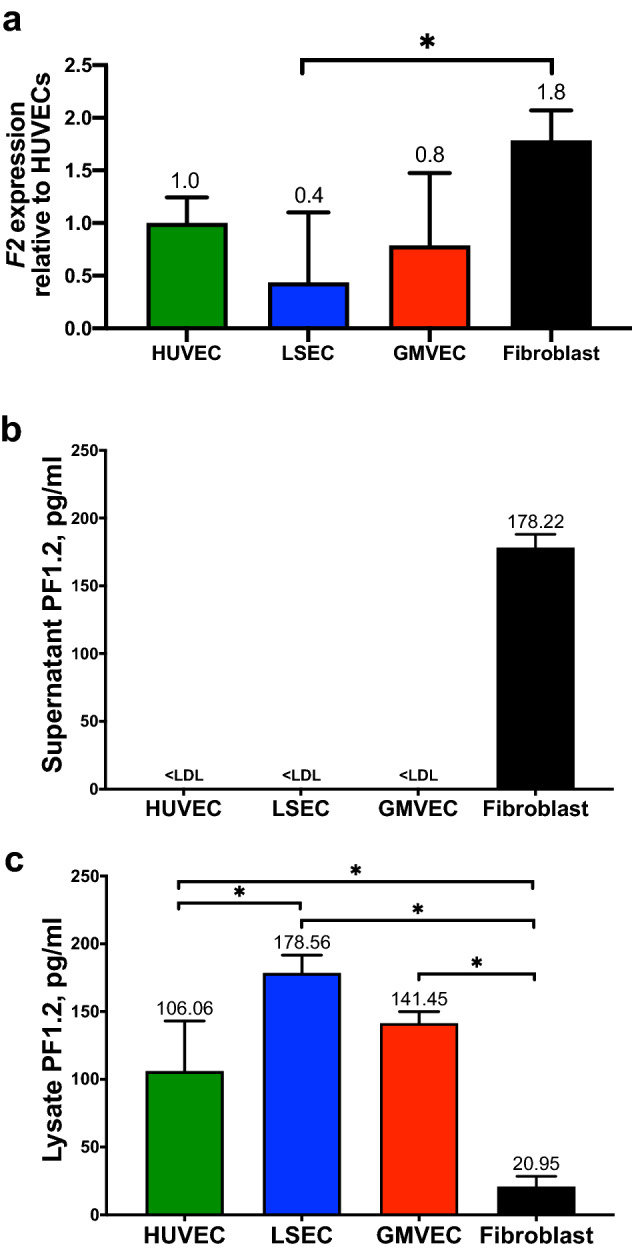
Gene expression of *F2* and PF1.2 levels in supernatant and lysate/cell membrane fractions of ECs and fibroblasts. (**a**) The *F2* expression levels in each cell type was quantified relative to *F2* levels in HUVECs after normalization to *GAPDH* in each real-time PCR analysis. Data shown are means + SD from four to six separate RNA extractions for each cell type (HUVEC, n = 6, LSEC, n = 6, GMVEC, n = 5, fibroblast, n = 4). Levels of PF1.2 were quantified in cell (**b**) supernatant and (**c**) lysates/cell membrane fractions of HUVECs (n = 3), LSECs (n = 3–4), GMVECs (n = 3), and fibroblasts (n = 3). PF1.2 concentrations (means + SD) in pg/ml were normalized to total lysate protein to account for cell number differences. Values below the lowest detectable limit of the assay are noted as < LDL. *p < 0.05.

Expression levels of *F2*, (FII gene) were measured from HUVECs, LSECs, GMVECs, and fibroblasts. Levels of *F2* in each cell type were quantified relative to the *F2* levels in HUVECs. The fibroblast *F2* mRNA levels are about twofold higher than *F2* levels in each EC type (Fig. 2a, fibroblasts and LSECs, *p* = 0.0038). The complete comparison statistics for HUVEC, LSEC, GMVEC, and fibroblast *F2* expression is shown in Supplementary Table S1. We previously found that the FII (prothrombin) protein levels in fibroblast lysate and supernatant were below the limit of detection in the assay we used^6^.

### PF1.2 protein quantification in supernatant and lysate/cell membrane fractions of ECs and fibroblasts

The calcium-dependent interaction of the vitamin K-dependent coagulation proteins with cell surface phospholipids is mediated by their γ-carboxyglutamate (GLA) domains^38,39^. Glutamic acid residues are converted to GLA residues by γ-glutamyl carboxylase in reactions requiring reduced vitamin K, carbon dioxide, and oxygen^40^. The PF1.2 portion of FII remains bound to cell surface phospholipids through these GLA domains following the cleavage of FII into PF1.2 and thrombin^41–45^.

**Figure 3:**
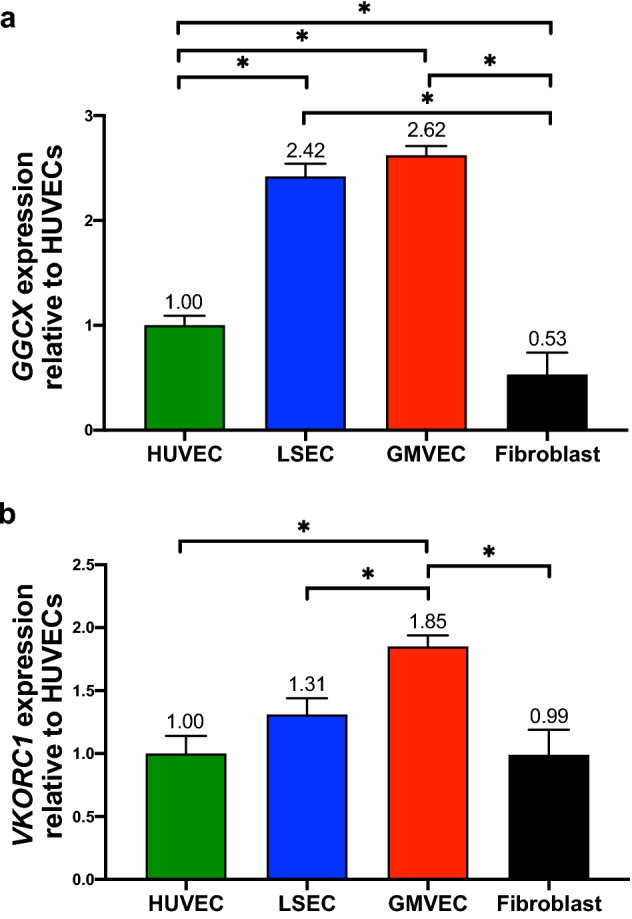
Gene expression of *GGCX* and *VKORC1* in ECs and fibroblasts. HUVEC, LSEC, GMVEC, and fibroblast expression levels of (**a**) *GGCX* (γ-glutamyl carboxylase) and (**b**) *VKORC1* (vitamin K epoxide reductase complex subunit 1) were analyzed by real-time PCR and quantified relative to gene expression levels in HUVECs. Data shown are means + SD from triplicate PCR measurements (n = 3 for each cell type). *p < 0.05.

PF1.2 levels were measured as a marker of prothrombinase activity in ECs and fibroblasts. There were detectable levels of PF1.2 in fibroblast though not EC supernatant (Fig. 2b). LSEC lysates contained 1.7-fold more PF1.2 than HUVECs (*p* = 0.044); and HUVEC, GMVEC, and LSEC lysates contained between 5- and 8.5-fold more PF1.2 than fibroblast lysates (*p* = 0.0024, 0.0002, and < 0.0001, respectively, Fig. 2c). The lower PF1.2 levels in fibroblast lysates than in EC lysates suggest the possibility that the number of GLA residues in amino-terminal domains on fibroblast-produced FII, and on PF1.2, are insufficient for stable cell anchoring on fibroblast surfaces. We then proceeded to conduct experiments on this possibility (Fig. 3).

### *GGCX* and *VKORC1* gene expression in ECs and fibroblasts

The gene expression levels of *GGCX* and *VKORC1*, the genes for γ-glutamyl carboxylase and vitamin K epoxide reductase complex subunit 1, respectively, were measured in HUVECs, LSECs, GMVECs, and fibroblasts. *GGCX* expression in fibroblasts was 2-, 4.5-, and 5-fold lower than HUVEC (*p* = 0.013), LSEC (*p* < 0.0001), and GMVEC (*p* < 0.0001, Fig. 3a) levels, respectively. *GGCX* expression was 2.4- and 2.6-fold higher in LSECs (*p* < 0.0001) and GMVECs (*p* < 0.0001, Fig. 3a) compared to HUVECs, respectively. Expression of *VKORC1* was 1.8-, 1.4-, and 2-fold higher in GMVECs compared to HUVECs (*p* = 0.0004), LSECs (*p* = 0.0081), and fibroblasts (*p* = 0.0004, Fig. 3b). The complete comparison statistics for HUVEC, LSEC, GMVEC, and fibroblast *GGCX* and *VKORC1* expression is shown in Supplementary Table S1. Lower γ-glutamyl carboxylase levels in fibroblasts compared to ECs presumably results in fibroblast-synthesized vitamin K-dependent coagulation proteins with fewer GLA residues and inefficient cell anchoring capacity. This presumption is substantiated by the results in Fig. 2b and c showing that the majority (~ 90%) of fibroblast-produced PF1.2 is found in the fibroblast supernatant instead of the lysate fraction containing fibroblast cell membranes.

In summary, we found that prothrombin fragment 1.2 was detected only in the lysate/cell membrane fractions of ECs and primarily (~ 90%) in the supernatant of fibroblasts. Fibroblast expression levels of *GGCX* (the carboxylase) were between two and fivefold lower than HUVEC, LSEC, and GMVEC levels, whereas *VKORC1* (the reductase) expression levels were elevated in GMVECs compared to other cell types, although similar between fibroblasts and HUVECs and LSECs. We surmise that the lower carboxylase expression levels in fibroblasts resulted in the production of FII with inefficient capacity to anchor to fibroblast membranes.

### Measurement of EC and fibroblast thrombin activities

Thrombin activity was measured using a thrombin-specific substrate in two different ways. In both experiments cells were maintained in serum-free media containing 5 mM CaCl_2_ for 24-h, and then washed prior to use. In the first measurements, thrombin activities were measured in lysate/cell membrane and supernatant fractions collected from HUVECs, LSECs, GMVECs, and fibroblasts. Samples were collected as for previous protein quantification except that lysates were collected without protease/phosphatase inhibitors in order to avoid thrombin neutralization. In the second measurements, thrombin activity was measured directly from live HUVECs and fibroblasts in culture plates with the addition of only the thrombin-specific substrate (S-2238) and appropriate calcium-containing buffer.

The thrombin activity measured in the lysate/cell membrane fraction of each cell type was similar, without significant differences (Table 1, upper portion). Conversely, each corresponding cell supernatant tested was negative for thrombin activity. The undetectable thrombin activity in cell supernatants likely resulted from the threefold increased volume in cell supernatants compared to cell lysate/cell membrane fractions. The increased volume is necessary to cover completely the cell surfaces during the 24-h prior to sample collection. The chromogenic thrombin substrate was not cleaved by 0.1 IU/ml thrombin in the presence of 1.78 U/ml antithrombin (AT) (plasma levels of AT, Table 1).

**Table 1:** Thrombin activity measurements.

	Thrombin activity, 10^–3^ IU/ml
**Cell lysate/membrane fraction**	
HUVECs	4.87 ± 0.78
LSECs	5.24 ± 1.68
GMVECs	4.64 ± 0.50
Fibroblasts	4.22 ± 0.44
IU/ml thrombin + 1.78 U/ml AT	< LDL
**Live cells**	
HUVECs	8.01 ± 0.72*
Fibroblasts	4.71 ± 1.17

Thrombin activities were calculated from cleavage rates of a thrombin-specific chromogenic substrate and converted into IU/ml. The values shown (mean ± SD) were calculated from samples with thrombin activities above the lower detection limit (LDL) of the assay (3.8 (10^–3^) IU/ml). Number of cell lysate samples with thrombin activities > LDL per total samples tested were HUVECs, 8/15; LSECs, 2/3; GMVECs, 3/3; and fibroblasts, 3/4. The thrombin activities measured in cell lysate/membrane fractions were not significantly different in each cell type according to Dunn’s multiple comparison test. In the live cell measurements, the thrombin activities measured on live HUVECs were 1.7-fold higher than live fibroblast thrombin activities (*HUVECs versus fibroblasts *p* < 0.0001). The thrombin activities were measured on live HUVECs (n = 6), on live fibroblasts (n = 11), and in thrombin standards (n = 4). Antithrombin (AT).

In more physiological experiments, the thrombin activities measured on live HUVECs were ~ twofold higher than measured activities on live fibroblasts (Table 1, lower portion, *p* < 0.0001). The thrombin activity was measured via the cleavage of a thrombin-specific chromogenic substrate (S-2238) in a calcium-containing buffer. Thrombin activity was measured over time, without the addition of external proteins or phospholipids, by changes in absorbance resulting from the thrombin-mediated cleavage of S-2238.

In summary, thrombin was generated and could be measured in EC and fibroblast lysate/cell membrane fractions. The measured thrombin activities were similar in all cell types. Thrombin was also generated and measured under more physiological conditions using live HUVECs and fibroblasts. In the more physiological experiments using live, intact cells, HUVECs produced almost twofold more thrombin than fibroblasts.

### Fluorescence colocalization

We previously reported colocalization of intracellular FII, FVII, FIX, and FX with Golgi and endoplasmic reticulum proteins in ECs indicating coagulation protein synthesis in these cells^6^. In this current study we report positive co-detection of FII/thrombin with thrombomodulin on HUVEC and GMVEC surfaces. FII/thrombin was detected in association with thrombomodulin with higher frequency on HUVEC surfaces (Supplemental Table S2). These observations support our hypothesis of prothrombinase complex formation and thrombin generation. The polyclonal sheep antibody made against human thrombin that was used in this study also detects FII. The fluorescent detection of FII/thrombin was low compared to thrombomodulin which is abundant on EC surfaces (Supplementary Fig. S2).

### Comparison of thrombomodulin on EC and fibroblast surfaces

**Figure 4:**
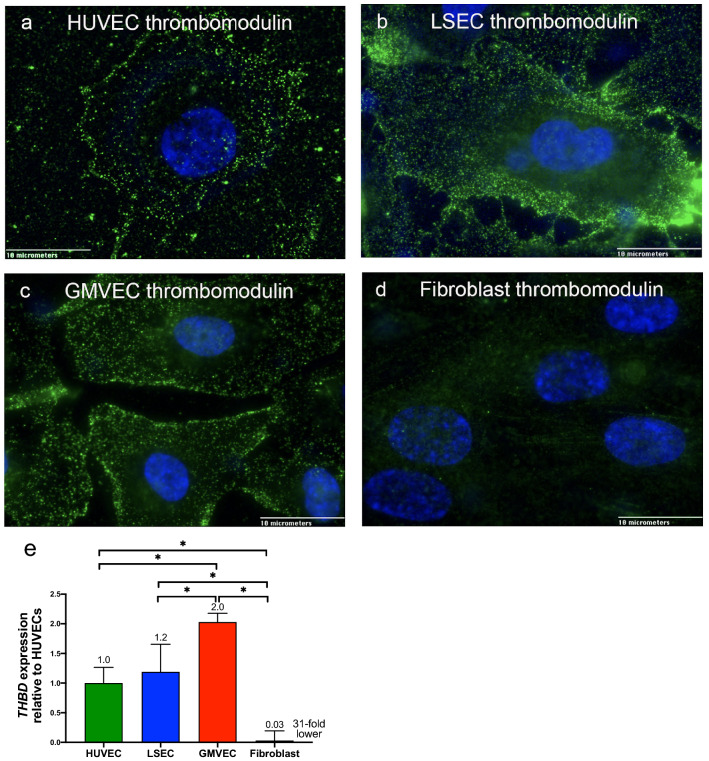
Immunofluorescent detection of thrombomodulin on surfaces of HUVECs, LSECS, GMVECs, and fibroblasts and *THBD* (thrombomodulin) gene expression. HUVECs (**a**), LSECs (**b**), GMVECs (**c**), and fibroblasts (**d**), were washed, treated with fixative, and stained with mouse anti-thrombomodulin + secondary goat anti-mouse IgG AF-488. The nuclei were stained with DAPI, and cells were imaged at 100X. Images were chosen from 10 to 30 microscopic fields using each cell type. (**e**) Expression levels of *THBD* in each cell type was quantified relative to *THBD* levels in HUVECs after normalization to *GAPDH* in each real-time PCR analysis. Data shown are means + SD from four to five separate RNA extractions for each cell type (HUVEC, n = 5, LSEC, n = 4, GMVEC, n = 4, fibroblast, n = 4). *p < 0.05.

Surfaces of HUVECs, LSECs, GMVECs, and fibroblasts were stained using antibodies to detect thrombomodulin plus secondary fluorescent antibodies. Images in Fig. 4, panels a–c, demonstrate the abundance of thrombomodulin on EC surfaces compared to fibroblasts in panel d.

### Expression levels of *THBD* (thrombomodulin gene) in ECs and fibroblasts

The *THBD* expression levels were ~ 30-fold higher in ECs than in fibroblasts (HUVECs *p* = 0.0012, GMVECs *p* < 0.0001, LSECs *p* = 0.0004, Fig. 4e). These results are in agreement with the immunofluorescent studies (Fig. 4a–d) showing the high level of thrombomodulin on EC surfaces and absence of thrombomodulin on fibroblast surfaces. In addition, *THBD* mRNA expression levels were higher in GMVECs compared to HUVECs (*p* = 0.0007) and LSECs (*p* = 0.0057, Fig. 4d). The complete comparison statistics for HUVEC, LSEC, GMVEC, and fibroblast *THBD* expression is shown in Supplementary Table S1.

In summary, FII/thrombin were co-detected with thrombomodulin on the surfaces of HUVECs and GMVECs. Surface thrombomodulin on ECs greatly exceeded FII/thrombin in immunofluorescence experiments. The fluorescent images demonstrate the copious amounts of thrombomodulin on the surfaces of HUVECs, LSECs, and GMVECs. Thrombomodulin was undetectable on fibroblast surfaces. Expression levels of *THBD* were ~ 30-fold higher in ECs than in fibroblasts.

### Gene expression of *SERPINC1* (antithrombin gene) in ECs and fibroblasts

**Figure 5:**
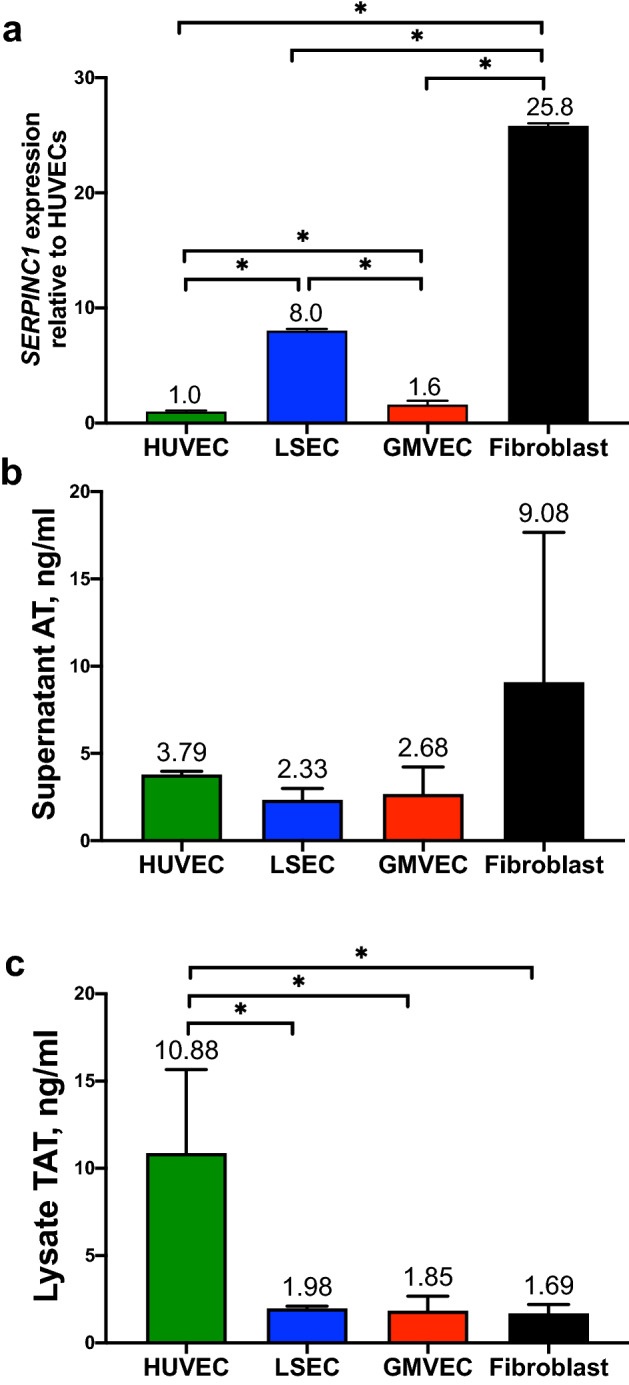
*SERPINC1* (antithrombin gene) expression and quantification of AT and TAT in ECs and fibroblasts. (**a**) The expression levels of *SERPINC1* in each cell type were analyzed by real time-PCR and quantified relative to HUVEC levels after normalization to *GAPDH* in each PCR analysis. Data shown are means + SD from three separate RNA extractions for each cell type. *p < 0.0001. (**b**) Levels of AT via ELISA were quantified in supernatant of HUVECs (n = 3), LSECs (n = 4), GMVECs (n = 3), and fibroblasts (n = 5). (**c**) Quantification of TAT in the lysate/cell membrane fraction of HUVECs, LSECs, GMVECs, and fibroblasts (n = 3 for each cell type). *p < 0.05.

Antithrombin binds and neutralizes thrombin by binding to the serine protease in a 1:1 ratio, forming TAT. Prior to analysis of TAT in ECs and fibroblasts, we determined whether these cells express and produced AT. We found that each cell type expressed *SERPINC1* and released AT into the supernatant. *SERPINC1* mRNA levels in fibroblasts were ~ 20-fold higher than EC levels (Fig. 5a, *p* < 0.0001). LSEC expression levels were eightfold higher than HUVEC and fivefold higher than GMVEC levels (*p* < 0.0001 for both cell types), and GMVEC expression levels were 1.6-fold higher than HUVECs (*p* = 0.041). The complete comparison statistics for HUVEC, LSEC, GMVEC, and fibroblast *SERPINC1* expression is shown in Supplementary Table S1.

### Quantification of AT in EC and fibroblast supernatant

Antithrombin levels could be measured in the supernatant, but not in the lysate/cell membrane fractions, of HUVECs, LSECs, GMVECs, and fibroblasts. Levels of AT were similar (with nonsignificant differences) in supernatants of each cell type (Fig. 5b).

### Quantification of TAT in ECs and fibroblasts

Protein levels of TAT were measured in supernatants and lysate/cell membrane fractions of ECs and fibroblasts, to provide evidence of FII cleavage to generate thrombin, followed by thrombin attachment to AT. The results in Fig. 5b show that AT is produced by ECs and fibroblasts and released into the supernatant. Contrary to the cell supernatant fraction containing AT protein, TAT was only detected in cell lysates. Concentrations of TAT in HUVEC lysates were ~ two to sevenfold higher than in LSEC (*p* = 0.009), GMVEC (*p* = 0.0082), and fibroblast (*p* = 0.0075) lysates, whereas cell types other than HUVECs had similar TAT levels (Fig. 5c).

To summarize, each cell type expressed *SERPINC1* and these mRNA levels in fibroblasts were ~ 20-fold higher than EC levels. Antithrombin protein levels were similar in the supernatants of ECs and fibroblasts but were not detected in either of the cell lysate/cell membrane fractions. In contrast to AT, TAT was only detected in cell lysate/cell membrane fractions. This indicated that some thrombin is attached to membrane AT (bound to heparan sulfate).

## Discussion

**Figure 6:**
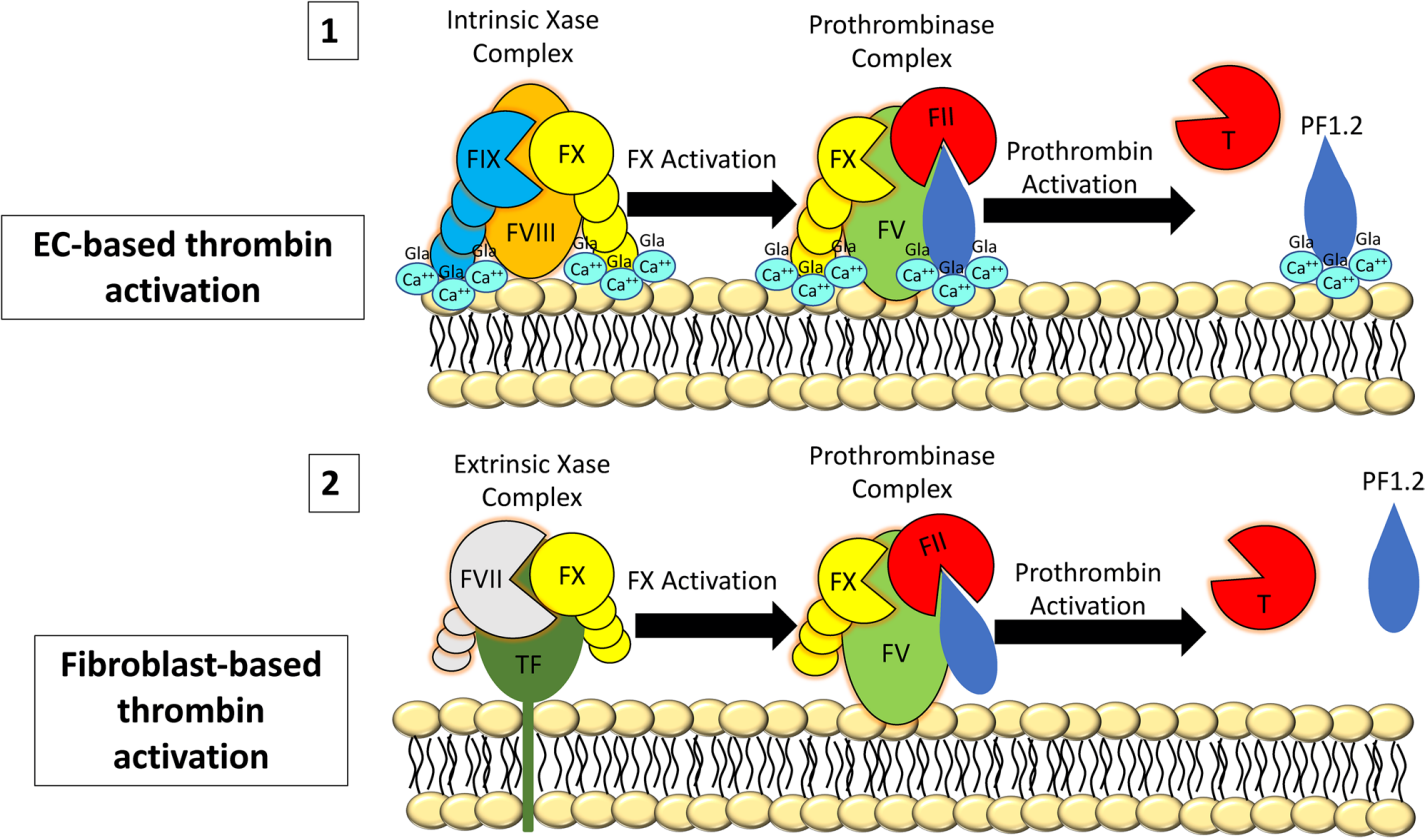
Visual representation of thrombin activation on the surfaces of ECs and fibroblasts. Coagulation reactions occur on the surfaces of both EC and fibroblasts with important differences between the cell types. These are: (1) EC-surface coagulation is likely initiated by the intrinsic Xase (FVIII–FIX) complex^6^; followed by stable prothrombinase formation. After FII activation, thrombin is released^18^, while PF1.2 remains bound through GLA-domains and Ca^++^ interactions with EC surfaces; and (2) fibroblast-surface coagulation is likely initiated by the extrinsic Xase (TF–FVII) complex. Transient prothrombinase complexes are formed, apparently with FV as the only coagulation component attached to fibroblast surfaces. Following FII activation, thrombin and the majority of PF1.2 is released from fibroblast surfaces. The reduced γ-carboxylation of fibroblast-produced coagulation factors is represented by shorter vitamin-K dependent coagulation factor “tails,” and the absence of GLA-Ca^++^ to indicate less surface interaction.

In this study, we have found that human ECs (HUVECs, GMVECs, and LSECs) and fibroblasts in culture produce the coagulation proteins required for prothrombinase complex formation. We also found that FII is activated on EC and fibroblast surfaces without the requirement of external coagulation proteins. Our experiments indicate that thrombin is generated by cell types that compose human vascular walls. We provide evidence that FII is activated, via the prothrombinase complex by our measurements of the FII cleavage product PF1.2, direct thrombin activity, and TAT. A visual representation of coagulation activity on EC and fibroblast surfaces is displayed in Fig. 6. These current results, when combined with our previous findings, that human ECs and fibroblasts produce the coagulation proteins necessary for FX activation on their surfaces^5,6^, provide evidence that cells of the human vascular wall participate in coagulation activation.

In addition to its role in hemostasis, thrombin has additional EC-specific functions. The thrombin–thrombomodulin complex on EC surfaces activates PC, which is an important anticoagulant reaction that limits hemostasis through the inactivation of FV and FVIII^21–23^. The thrombin activity measured directly in human ECs (Table 1) is likely to represent only a portion of the total amount of thrombin produced by ECs. A considerable quantity of EC-produced thrombin may be bound by AT and thrombomodulin (limiting thrombin-induced fibrinogen cleavage to fibrin polymers)^46^. See a cartoon summary of these processes in Supplementary Figure S3. Although both fibroblasts and ECs produce AT to bind thrombin, only ECs produce abundant quantities of thrombomodulin which binds thrombin with high affinity^47^. Thrombomodulin was absent on fibroblast surfaces in immunofluorescent studies, and mRNA levels of *THBD* (thrombomodulin gene) were 30-fold higher in ECs than in fibroblasts (Fig. 4).

FV is an integral component of the prothrombinase complex (FX–FV–FII) after FX is activated by FVIII–FIX (intrinsic pathway) or by TF–FVII (extrinsic pathway). Our results show that ECs and fibroblasts both express the mRNA for FV and produce FV protein. FV attaches to cell surfaces utilizing their discoidin domains, C1 and C2^48^. It has also been suggested that the A3 domain of FV interacts with phospholipids and may insert into the cell^34^. The surface FV on both cell types was capable of active participation in prothrombinase complex formation that resulted in thrombin production. *F5* mRNA levels were 20-fold higher in ECs than in fibroblasts. Contrary to the expression data, however, FV protein levels were similar in supernatants of both cell types and were two to fourfold higher in fibroblast lysates than in EC lysates (Fig. 1). The probable explanation for this finding is that FV is continuously inactivated by activated PC (APC)–thrombomodulin on EC surfaces and requires constant replenishment. In contrast, fibroblasts have little or no thrombomodulin, and do not have thrombin–thrombomodulin–APC to cleave and inactivate FV (Fig. 4).

Our experiments reported in this manuscript demonstrate that FII is activated on fibroblasts, as well as on ECs. Fibroblasts have higher levels of FV than ECs (Fig. 1b,c), produce > 1000-fold greater amounts of TF, and release forms of FVII and FX capable of “extrinsic” FX activation^6^. In our previous study^6^, we found that fibroblasts were able to activate only minimal amounts of FX without the addition of GLA-containing forms of FVII and FX purified from human plasma.

Fibroblast mRNA levels of *F2* were ~ twofold higher than EC levels (Fig. 2a); however, FII was undetectable in both fibroblast supernatant and lysates^6^. In contrast, similar quantities of FII were measured in all 3 types of EC lysates^6^. The fibroblast levels of PF1.2 (Fig. 2b,c) and the previously reported^6^ undetectable levels of FII appear initially to be inconsistent. These seemingly contrary measurements are the result of differences in the lower detection limits in these two assays. The PF1.2 assay has a lower detection limit (LDL) of 28 pg/ml and is more sensitive than the FII assay that has an LDL of 188 pg/ml. The measured PF1.2 level of 178 pg/ml in fibroblast supernatants was just below the LDL of the FII assay. This result suggests that the FII levels in fibroblast supernatants are probably similar to PF1.2 levels in fibroblast supernatants, and therefore, fibroblasts can promote prothrombinase complex formation (Table 2). We suspect that the fibroblast FII is synthesized without the necessary GLA-containing domains for stable cell surface attachment (Fig. 3). Fibroblast FII production, possibly with deficient γ-carboxylation, must be sufficient to allow for some interaction with FX and FV, for fibroblasts to generate thrombin (Table 1) and PF1.2 (Fig. 2b,c).

**Table 2:** Comparison of vitamin K-dependent coagulation protein levels in the supernatant and lysate/cell membrane fractions of ECs and fibroblasts.

Vitamin K-dependent proteins	ECs	Fibroblasts
FII supernatant	< LDL of 188 pg/ml	< LDL of 188 pg/ml
FII lysate	245–287 pg/ml	< LDL of 188 pg/ml
PF1.2 in supernatant*	< LDL of 28 pg/ml	178 pg/ml
PF1.2 in lysate*	106–179 pg/ml	21 pg/ml, ~ fivefold lower
FVII supernatant	459–1667 pg/ml	647 pg/ml
FVII lysate	69–428 pg/ml	43 pg/ml
FIX supernatant	10–13 ng/ml	12 ng/ml
FIX lysate	5–15 ng/ml	3 ng/ml, two to fivefold lower
FX supernatant	379–475 pg/ml	2571 pg/ml, five to sevenfold higher
FX lysate	731–1694 pg/ml	1136 pg/ml

Protein levels of the vitamin K-dependent coagulation proteins in the supernatant and lysate/cell membrane fractions of ECs (HUVEC, GMVEC, LSEC) and fibroblasts. There are higher concentrations of these proteins in EC lysates than in fibroblast lysates.

*Values from the current study. The other values are from Cohen et al.^6^.

We provide evidence in this study of decreased lysate/cell membrane fraction binding of vitamin K-dependent coagulation proteins on fibroblasts compared to ECs. Fibroblast supernatant has higher concentrations of PF1.2 (Fig. 2b) and FX than HUVECs^6^, whereas fibroblast lysates have lower amounts of PF1.2 and FX than HUVECs. Furthermore, similar levels of FVII and FIX were measured in fibroblast and HUVEC supernatants despite HUVEC lysate containing tenfold more FVII and fivefold more FIX than in fibroblast lysate^6^. These findings all suggest that a high proportion of PF1.2, FVII, FIX, and FX produced by fibroblasts are directly released with minimum binding to fibroblast surfaces. In ECs, higher amounts of the vitamin K-dependent proteins FII and FX and the FII activation product PF1.2 were measured within the lysate/cell membrane fractions compared to supernatant (Table 2).

The decreased fibroblast lysate/cell surface binding likely results from reduced activity of the fibroblast γ-glutamyl carboxylase^49^. The NH_2_-terminus of the PF1.2 portion of FII, like the other vitamin K-dependent coagulation proteins, binds to cell surface phospholipids through GLA-rich domains (~ 9–12 GLA) in a calcium-dependent manner. Glutamic acid residues are converted into GLA residues in the endoplasmic reticulum by γ-glutamyl carboxylase. This reaction occurs in conjunction with vitamin K epoxide reductase. We found that mRNA levels for the carboxylase (*GGCX*) were two to fivefold lower in fibroblasts than in ECs, whereas mRNA levels of the reductase (*VKORC1*) were similar in fibroblasts and ECs, although GMVEC levels were twofold higher (Fig. 3). Studies using γ-carboxyl-glutamate-deficient FII have shown that the reduction of only 3 GLA residues may reduce phospholipid binding affinity by 1000-fold, demonstrating and the importance of complete FII carboxylation for proper membrane binding^49–52^. Therefore, the two to fivefold reduced *GGCX* expression in fibroblasts must have considerable impact on fibroblast γ-carboxylation activity.

In conclusion, our findings demonstrate that cell types that comprise the human vascular wall, ECs and fibroblasts, both form active prothrombinase complexes on their surfaces using distinct processes. Prothrombinase complexes on EC surfaces (FX–FV–FII) include membrane-attached FV and are further stabilized through interactions with phospholipids on EC membranes by calcium-dependent GLA-domains of FX and FII. On fibroblast surfaces, the prothrombinase complexes consist of membrane-attached FV and transient interactions with γ-carboxy-glutamate-deficient forms of FX and FII. This study contributes additional evidence that ECs and fibroblasts of the vascular wall have an active role in hemostasis.

## Materials and methods

### Human endothelial cells

Human umbilical vein endothelial cells (HUVECs) were purchased as pooled primary cells from Cell Applications Inc. (200p-05n). Glomerular microvascular endothelial cells (GMVECs) (ACBRI-128) and liver sinusoidal microvascular endothelial cells (LSECs) (ACBRI-566) were isolated from single donors and purchased from Cell Systems. ECs were grown in MCDB-131 (Sigma-Aldrich) plus PSQA (120 units/ml penicillin; 100 µg/ml streptomycin; 2 mM l-glutamine; 0.25 µg/ml amphotericin) and low serum growth supplement (S003K, fetal bovine serum [FBS] concentration 2% v/v) for HUVECs, or microvascular growth supplement (S00525, 5% FBS, v/v) for LSECs and GMVECs. For passage, ECs were non-enzymatically removed from tissue culture flasks using 5 mM EDTA in Ca^+2^/Mg^+2^-free PBS and cell scraping. Each EC type was used in experiments at passages 3–8 derived from a single lot.

### Human dermal fibroblasts

Single donor human fibroblasts were purchased from Cell Applications Inc. (106-05a) at passage 2. Fibroblasts were grown in basic growth media MCDB-131 plus PSQA and low serum growth supplement (S003K, 2% FBS, v/v), and used in experiments at passages 3–8.

### Protein measurements

#### Endothelial cell and fibroblast lysate/cell membrane fractions

Cell lysates (containing solubilized cell membranes) were prepared from HUVECs, GMVECs, LSECs, and fibroblasts grown in T-75 and T-25 flasks until confluence. Cells were washed 3X with Ca^+2^/Mg^+2^-containing PBS, and then the media was replaced with serum-free medium (MCDB-131 + insulin [10 mg/l]-transferrin [5.5 mg/ l]-selenium [6.7 µg/ l], Life Technologies) containing 5 mM CaCl_2_ for 24 h (500 µl per T-25 flask and 1.5 ml per T-75 flask). On collection day, cells were washed 3X with 10 ml (T-75) or 4 ml (T-25) of cold, sterile Tris, pH 7.3 buffer (50 mM Tris, 1% BSA, and 5 mM CaCl_2_) to remove any exogenous proteins. Cells in T-75 flasks were lysed with 500 µl of CelLytic M (ice cold, Sigma-Aldrich C-2978) + 10 µl of Halt protease/phosphatase inhibitor cocktail (Thermo Scientific, 78,430) for 15 min with rocking. T-25 flasks required 175 µl of CelLytic M + 3 µl of inhibitor cocktail. Lysates used for thrombin activity measurement were prepared without the inhibitor cocktail to preserve thrombin activity. Lysed cells were collected with cell scrapers, placed into chilled tubes, and centrifuged at 12,000 g for 15 min at 4 °C. The soluble fractions were collected and stored at − 80 °C until used for specific protein quantification assays.

#### Endothelial cell and fibroblast supernatant

EC and fibroblast supernatants were collected after 24-h in serum-free media containing 5 mM CaCl_2_ (1.5 ml/T-75 flask and 500 µl/T-25 flask), into tubes containing 20% immunoglobin-free bovine serum albumin (BSA) to produce a final concentration of 1% BSA. Samples were stored at − 80 °C until used for specific protein quantification assays.

#### Quantification of FV, AT, PF1.2, and TAT in EC and fibroblast lysate/cell membrane fractions and supernatant

The protein concentrations of FV, AT, PF1.2, and TAT were measured in EC and fibroblast undiluted lysates and supernatants. Proteins were quantified using purchased kits for FV (Assaypro, EF1005-1), PF1.2 (LSBio, LS-23736), and Abcam assays for AT (ab108801) and TAT (ab108907). The antibodies in the kits for FV, AT, and TAT only detect human proteins, without cross-reactivity with bovine proteins. The manufacturer states that no significant cross-reactivity or interference has been observed between the antibody in the PF1.2 kit and other FII/thrombin analogs. The lower detection limits (LDL) of the ELISA systems used were: FV = 72 pg/ml; AT = 0.93 ng/ml; PF1.2: = 28.13 pg/ml; and TAT = 0.84 ng/ml. Sample mean values below these limits were denoted as < LDL. Values obtained for FV, PF1.2, and TAT from lysate/cell membrane fractions were normalized to total lysate protein (in order to account for cell number differences) determined by the Bradford method (as Coomassie brilliant blue G-250 binds to proteins, absorbance at 595 nm increases proportionally to the amount of protein).

#### Reverse transcription real-time (RT) quantitative polymerase chain reaction (qPCR)

Cells were washed and maintained in serum-free media for 24 h prior to RNA extraction. RNA was isolated using TRIzol, chloroform extraction, and isopropanol precipitation. RNA integrity was verified by 1%-agarose-formaldehyde electrophoresis and 260/280 optical ratios. RNA reverse transcription was performed using SuperScript VILO MasterMix (Invitrogen). The resulting cDNA samples (100 ng) were amplified in triplicate by RT-qPCR under the conditions: 95 °C for 3 min, 50 cycles of (10 s at 95 °C, 10 s at 55 °C, 30 s at 72 °C), and 95° for 10 s (CFX96, BioRad). The amplified cDNA products were analyzed using TaqMan Gene Expression Assays (with 6-carboxy-fluorescein-labeled probes that span target exon junctions, except for *VKORC1* and *THBD* which does not have introns^53^) and PerfeCT FastMix II (Quanta).

### Quantitative relative gene expression measurements

The quantification of *F2*, *F5*, *SERPINC1* (antithrombin), *THBD* (thrombomodulin), *GGCX* (γ-glutamyl carboxylase), and *VKORC1* (vitamin K epoxide reductase complex subunit 1) gene expression in LSECs, GMVECs, and fibroblasts relative to expression levels in HUVECs was calculated as described by Livak and Schmittgen^54^. The standard deviation in gene expression assays (SD) was determined by the equation: SD = square root (S_1_^2^ + S_2_^2^) where S_1_ and S_2_ are means of the standard deviations of triplicate C_q_ measurements for the reference (*GAPDH)* and target genes.

### Measurement of thrombin activities in lysate/cell membrane and supernatant fractions of ECs and fibroblasts

HUVEC, GMVEC, LSEC, and fibroblast lysate/cell membrane fractions were collected without the use of the protease/phosphatase inhibitor cocktail. Thrombin activity was measured in 10 µl samples of undiluted lysate/cell membranes (7 µl lysis buffer/cm^2^ cell surface area) and supernatant fractions (20 µl SF-media/cm^2^ cell surface area) using the Abcam thrombin activity assay (ab234620). The cleavage of a specific thrombin substrate releases a *p*-nitroaniline chromophore that is detected at 405 nm. The change in mean 405 nm absorbance over time (measured every 30 min for 2 h) was recorded and cleavage rates were calculated. The minimum detectable value of human thrombin activity was stated by the manufacturer to be 0.031 AU/ml (0.0038 IU/ml). Samples below this threshold were denoted as negative. The manufacturer also reported that no significant cross-reactivity or interference has been observed with the thrombin-specific substrate. The specificity of the provided thrombin substrate was further tested by measuring the cleavage rate of 0.1 IU/ml thrombin after the addition of 1.78 U/ml AT (human antithrombin III, HCATIII-0120, Haematologic Technologies).

### Measurement of thrombin activity on live HUVEC and fibroblast surfaces

HUVECs and fibroblasts were grown to confluence in 12-well plates and washed 3× with Ca^+2^/Mg^+2^-containing PBS before replacement with serum-free media for 24 h. Before measurements, cells were washed 3× with Tris, pH 7.3 buffer (50 mM Tris, 1% BSA, and 5 mM CaCl_2_). To each well (4 cm^2^), 150 µl of Tris, pH 8.3 buffer was added (50 nM Tris, pH 8.3, 0.2% BSA, and 5 mM CaCl_2_) followed by 100 µl of the thrombin-specific chromogenic substrate S-2238 (Diapharma). Absorbance was measured at 405 nm and 490 nm in a Tecan Infinite M200 Pro plate reader every 15 min for 180 min. The A_490_ was subtracted from the A_405_ to correct for differences in the wells. The thrombin substrate composed of 25 mg of S-2238 dissolved in 7.14 ml sterile water to make a 5.6 mM stock solution, in accordance with instructions from the manufacturer for measuring thrombin activity.

### Thrombin activity calculations for live cell measurements

Human alpha thrombin (HCT-0020, Haematologic Technologies) was diluted in 50 mM Tris, pH 8.3, 1% BSA, and 5 mM CaCl_2_ to concentrations ranging from 1000 to 15 pM with activities of 125 (10^−3^) to 2 (10^−3^) IU/ml. S-2238 was added to the thrombin standards (in the same Tris buffer at pH 8.3) retaining the same volume ratio as in the experiments measuring thrombin activities on the live cells grown in 12-well plates. Absorbance at 405 nm was recorded every 5 min for 60 min. Equations generated from plots of the slopes (cleavage rates) versus thrombin activity (IU/ml) were used to calculate the thrombin activity levels in the live cell experiments. The cleavage rate of each thrombin standard was measured 4 times.

### Fluorescent microscopy studies

#### Microscope image acquisition

Our microscope system consists of a Nikon Diaphot TE300 microscope equipped with CFI Plan Fluor 60 × oil, numerical aperture (NA) 1.4 and CFI Plan Apo Lambda 100 × oil, NA 1.45 objectives, a 10 × projection lens and a Prior motorized stage. Fluorescent images were obtained with a SensiCamQE CCD camera (Cooke) using dual filter wheels (Prior) with single band excitation and emission filters for FITC/TRITC/CY5/DAPI (Chroma). Cell images were processed and analyzed using IP Lab software version 3.9.4r4 with a fluorescence colocalization module (Scanalytics). Images acquired using the 60 × objective have dimensions of 78 µm × 58 µm, and images acquired using the 100 × objective are 41 µm × 30 µm. Calibration bars on images are 10 µm.

#### Cell surface membrane fluorescent staining

Coagulation proteins on surface membranes of HUVECs, GMVECs, and fibroblasts were detected using fluorescent staining and microscopy. Cells were grown on gelatin-coated glass coverslips, and were washed with Tris, pH 7.3 buffer prior to fixation and before each antibody addition. Cells were fixed with 1% *p*-formaldehyde/Tris, pH 7.3 buffer for 10 min and stained with each primary antibody (diluted 1:100 in PBS containing 1% BSA) plus fluorescent-labeled secondary antibody at 20 µg/ml for 15 min. Cell nuclei were detected with DAPI (4′,6-diamidino-2-phenylindole, 1.5 µg/ml) that was included in the mounting medium (Fluoro-Gel II).

#### Antibody pairs for detection of cell surface proteins

Sheep anti-human FV (PA1-43041, Invitrogen) plus rabbit anti-sheep IgG Dylight-594 (SA5-10056, Invitrogen); Mouse anti-human thrombomodulin (MA1-90642, clone QBEND-40, ThermoFisher) plus goat anti-mouse IgG Alexa Fluor (AF)-488 (A11029, Invitrogen); Sheep anti-human thrombin (PAHT-S, also detects FII, Haematologic Technologies) plus donkey anti-sheep IgG AF-647 (A21448, Invitrogen).

### Statistical analysis

GraphPad Prism v 9.2 software (GraphPad Software Inc., San Diego, CA) was used for ELISA analysis, to calculate significance of differences between experimental results using one- and two-way ANOVAs and Tukey’s and Dunnett’s multiple comparison tests with an alpha value of 0.05. The SD values in the fluorescent colocalization measurements were calculated using Microsoft Excel.

## Supplementary Information

Supplementary Information.

## Data Availability

The datasets generated during and/or analyzed during the current study are available from the corresponding author on reasonable request.
