# Supplementary material for: Human endothelial cells and fibroblasts express and produce the coagulation proteins necessary for thrombin generation
Source: Sci Rep. 2021 Nov 8;11:21852. doi: 10.1038/s41598-021-01360-w (PMC8575941; doi:10.1038/s41598-021-01360-w)
Supplement: Supplementary file 1 — Supplementary Information. [file 41598_2021_1360_MOESM1_ESM.pdf]

Human endothelial cells and fibroblasts express and produce the coagulation proteins necessary for thrombin generation

Clay T. Cohen, Nancy A. Turner, Joel L. Moake

Supplementary Material

Supplementary Table S1. Gene expression p-value summary

F5	Mean Diff.	95.00% CI of diff.	Summary	Adjusted P Value
HUVECs vs. LSECs	0.4558	0.04262 to 0.8689	*	0.0284
HUVECs vs. GMVECs	-0.1979	-0.6556 to 0.2598	ns	0.6087
HUVECs vs. Fibroblasts	0.9575	0.4998 to 1.415	*	0.0001
LSECs vs. GMVECs	-0.6537	-1.094 to -0.2133	*	0.0033
LSECs vs. Fibroblasts	0.5017	0.06127 to 0.9421	*	0.0232
GMVECs vs. Fibroblasts	1.155	0.6729 to 1.638	*	<0.0001

F2	Mean Diff.	95.00% CI of diff.	Summary	Adjusted P Value
HUVECs vs. LSECs	0.5629	-0.2721 to 1.398	ns	0.2607
HUVECs vs. GMVECs	0.213	-0.6959 to 1.122	ns	0.9098
HUVECs vs. Fibroblasts	-0.7843	-1.753 to 0.1845	ns	0.1381
LSECs vs. GMVECs	-0.35	-1.229 to 0.5289	ns	0.6791
LSECs vs. Fibroblasts	-1.347	-2.288 to -0.4065	*	0.0038
GMVECs vs. Fibroblasts	-0.9973	-2.004 to 0.009583	ns	0.0527

GGCX	Mean Diff.	95.00% CI of diff.	Summary	Adjusted P Value
HUVEC vs. LSEC	-1.42	-1.777 to -1.063	*	<0.0001
HUVEC vs. GMVEC	-1.62	-1.977 to -1.263	*	<0.0001
HUVEC vs. Fibroblast	0.47	0.1127 to 0.8273	*	0.0126
LSEC vs. GMVEC	-0.2	-0.5573 to 0.1573	ns	0.3428
LSEC vs. Fibroblast	1.89	1.533 to 2.247	*	<0.0001
GMVEC vs. Fibroblast	2.09	1.733 to 2.447	*	<0.0001

VKORC1	Mean Diff.	95.00% CI of diff.	Summary	Adjusted P Value
HUVEC vs. LSEC	-0.31	-0.6903 to 0.07026	ns	0.1152
HUVEC vs. GMVEC	-0.85	-1.230 to -0.4697	*	0.0004
HUVEC vs. Fibroblast	0.01	-0.3703 to 0.3903	ns	0.9998
LSEC vs. GMVEC	-0.54	-0.9203 to -0.1597	*	0.0081
LSEC vs. Fibroblast	0.32	-0.06026 to 0.7003	ns	0.1023
GMVEC vs. Fibroblast	0.86	0.4797 to 1.240	*	0.0004

THBD	Mean Diff.	95.00% CI of diff.	Summary	Adjusted P Value
HUVECs vs. LSECs	-0.1893	-0.7546 to 0.3759	ns	0.7613
HUVECs vs. GMVECs	-1.028	-1.594 to -0.4631	*	0.0007
HUVECs vs. Fibroblasts	0.9685	0.4033 to 1.534	*	0.0012
LSECs vs. GMVECs	-0.839	-1.435 to -0.2432	*	0.0057

LSECs vs. Fibroblasts	1.158	0.5620 to 1.754	*	0.0004
GMVECs vs. Fibroblasts	1.997	1.401 to 2.593	*	<0.0001

SERPINC1	Mean Diff.	95.00% CI of diff.	Summary	Adjusted P Value
HUVEC vs. LSEC	-7.04	-7.625 to -6.455	*	<0.0001
HUVEC vs. GMVEC	-0.61	-1.195 to -0.02501	*	0.0413
HUVEC vs. Fibroblast	-24.82	-25.40 to -24.24	*	<0.0001
LSEC vs. GMVEC	6.43	5.845 to 7.015	*	<0.0001
LSEC vs. Fibroblast	-17.78	-18.36 to -17.20	*	<0.0001
GMVEC vs. Fibroblast	-24.21	-24.79 to -23.63	*	<0.0001

Supplemental Table S1. Expression levels of *F5*, *F2*, *GGCX*, *VKORC1, THBD,* and *SERPINC1* were measured from HUVECs, LSECs, GMVECs, and fibroblasts. Gene expression p-values were calculated using Tukey's multiple comparisons test; Confidence interval (CI); Non-significant (ns); *p<0.05.

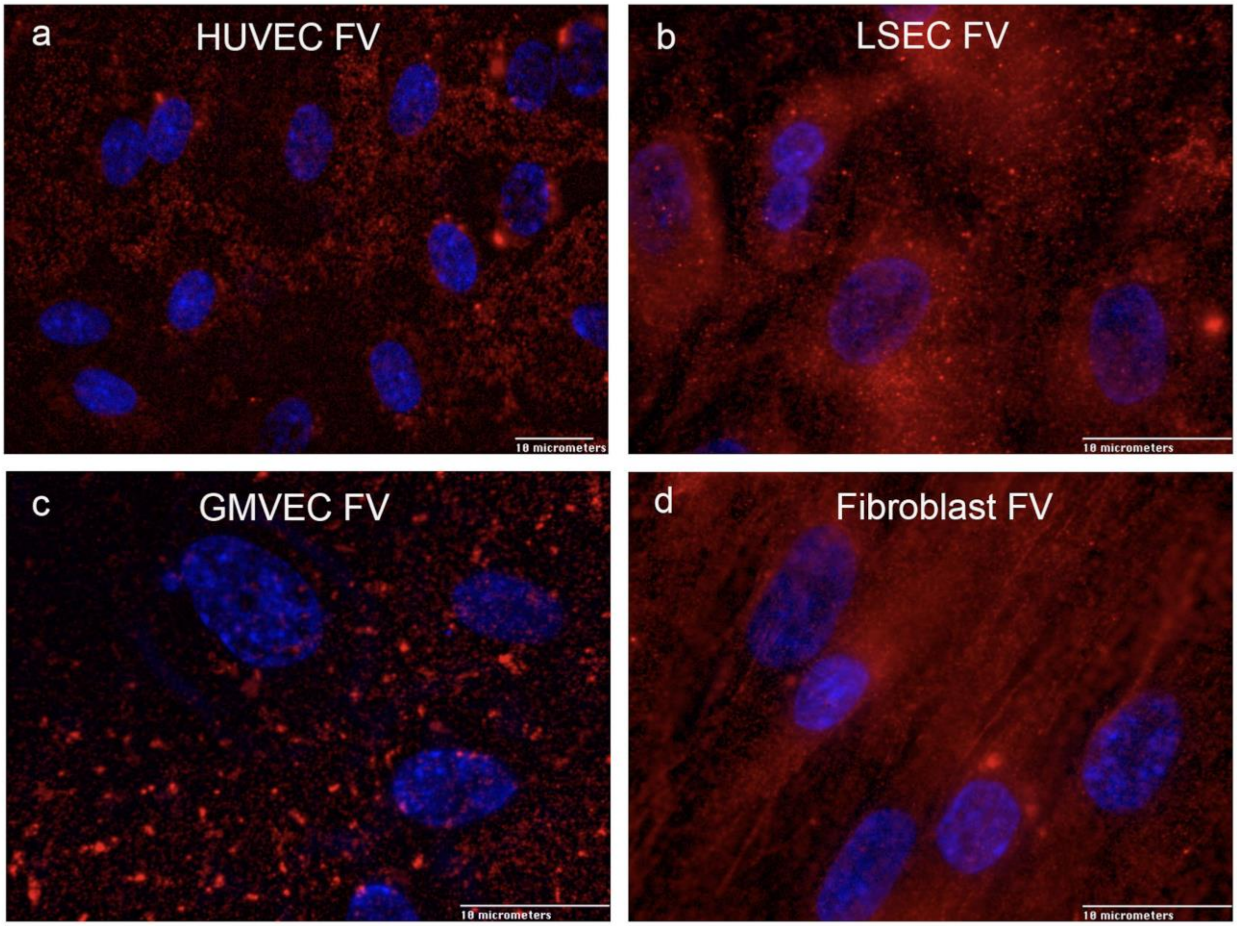

Supplementary Figure S1. Immunofluorescent detection of FV on surfaces of HUVECs, LSECs, GMVECs and fibroblasts. HUVECs (a), LSECs (b), GMVECs (c), and fibroblasts (d), were washed, treated with fixative and stained with sheep anti-human FV + secondary rabbit anti-sheep IgG Dylight-594. The nuclei were stained with DAPI and cells were imaged at 60X in (a) and at 100X in (b), (c) and (d). Images were chosen from over 30 microscopic fields using each cell type.

Supplemental Table S2. Thrombin and thrombomodulin colocalization coefficients

	HUVECs	GMVECs
	Thrombin* and Thrombomodulin, mean ± SD (range)	Thrombin* and Thrombomodulin, mean ± SD (range)
PC	0.55 ± 0.23 (0.14-0.85)	0.26 ± 0.09 (0.11-0.54)
OC	0.74 ± 0.12 (0.43-0.92)	0.65 ± 0.06 (0.53-0.79)
M1	0.78 ± 0.15 (0.46-0.97)	0.55 ± 0.20 (0.18-0.98)
M2	0.79 ± 0.16 (0.44-0.99)	0.48 ± 0.25 (0.11-0.99)
	n = 7, 35 images	n = 5, 41 images

Supplemental Table S2**.** Positive colocalization of FII/thrombin with thrombomodulin on EC surfaces. Colocalization coefficients, Pearson's (PC), Manders' Overlap (OC) and Manders' split signal coefficients (M1 and M2), were measured in HUVEC and GMVEC images stained concurrently with antibody pairs detecting thrombin/FII and thrombomodulin. The entire microscopic image at 100X was used to analyze the extent of co-localization between the proteins using detecting antibodies: sheep anti-human thrombin plus donkey anti-sheep IgG AF-647; and mouse anti-human thrombomodulin plus goat anti-mouse IgG AF-488. *The sheep polyclonal antibody recognizes FII and thrombin.

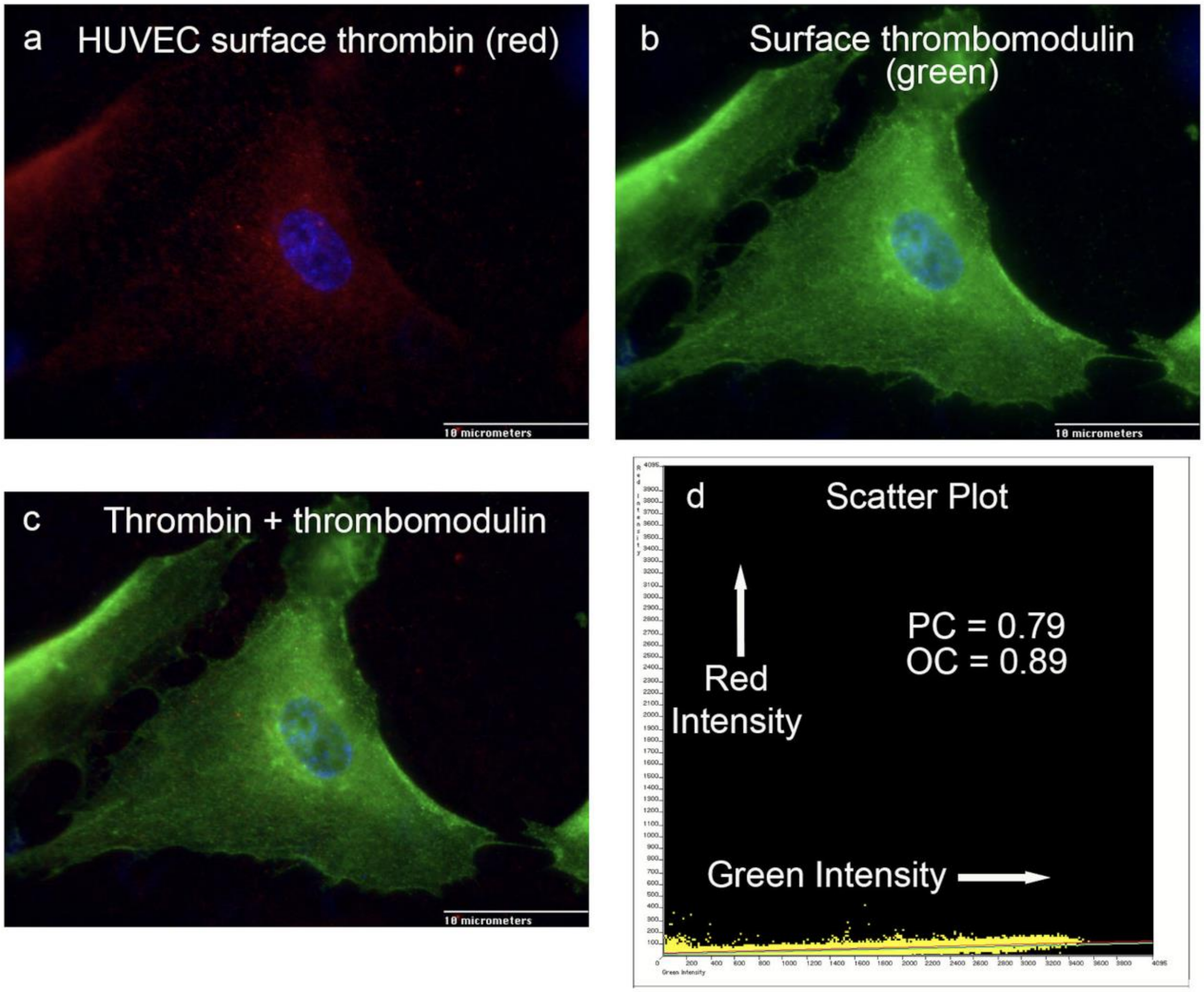

Supplementary Figure S2. Thrombin and thrombomodulin have positive colocalized detection on HUVEC surfaces. HUVECs were washed and treated with fixative prior to staining with antibodies detecting human thrombin/FII and thrombomodulin. (a) Surface detection of thrombin using sheep anti-thrombin/FII + donkey anti-sheep IgG AF-647. (b) Surface thrombomodulin detection using mouse anti-thrombomodulin + goat anti-mouse IgG AF-488. (c) Merged image of (a) and (b) detecting both thrombin/FII and thrombomodulin. (d) Scatter plot of the red intensity of thrombin versus the green intensity of thrombomodulin. Both colocalization coefficient values (PC = Pearson's coefficient and OC = Overlap coefficient) are approaching 1, indicating positive colocalized detection of thrombin with thrombomodulin. In (c), the high green intensity from the substantial amount of thrombomodulin present on HUVEC surfaces, overwhelms the red intensity of thrombin detection resulting in a mostly green image; and

in (d) this skewed ratio is reflected as almost a flat line along the x-axis (green intensity). The HUVECs were imaged at 100X and the nuclei were stained with DAPI.

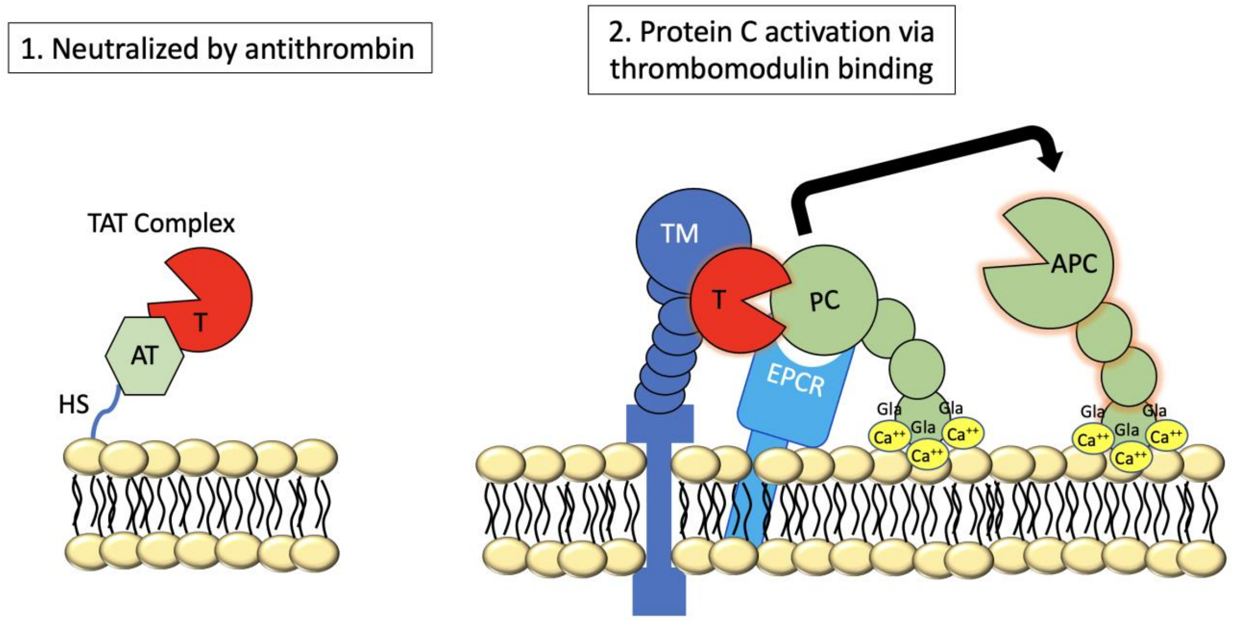

Supplementary Figure S3. The fate of thrombin following activation on cell surfaces. (1) On both ECs and fibroblasts, thrombin (T) is bound and neutralized by membrane AT (bound to heparan sulfate [HS]), resulting in TAT formation; and (2) on ECs, but not on fibroblasts, T binds to thrombomodulin (TM) and cleave/activates protein C (PC) (bound to endothelial protein C receptor [EPCR]), resulting in protein C activation (APC).

Supplemental Materials and Methods

Cell surface membrane fluorescent staining

FV protein on surface membranes of HUVECs, GMVECs and fibroblasts was detected using fluorescent staining and microscopy. Cells were grown on gelatin-coated glass coverslips, and were washed with Tris, pH 7.3 buffer (50 mM Tris, pH 7.3, 1% BSA, and 5 mM $CaCl_2$), prior to fixation and before each antibody addition. Cells were fixed with 1% p-formaldehyde/Tris, pH 7.3 buffer for 10 min and stained with sheep anti-human FV (PA1-43041, diluted 1:100 in PBS containing 1% BSA) plus fluorescent-labeled secondary rabbit anti-sheep IgG Dylight-594 (SA5-10056) at 20 µg/ml for 15 min. Cell nuclei were detected with DAPI (4',6-diamidino-2-phenylindole, 1.5 µg/ml) that was included in the mounting medium (Fluoro-Gel II).

Antibody pairs for detection of cell surface proteins

Sheep anti-human FV (PA1-43041, Invitrogen) plus rabbit anti-sheep IgG Dylight-594 (SA5-10056, Invitrogen)

Mouse anti-human thrombomodulin (MA1-90642, clone QBEND-40, ThermoFisher) plus goat anti-mouse IgG AF-488 (A11029, Invitrogen)

Sheep anti-human thrombin (PAHT-S, also detects FII, Haematologic Technologies) plus donkey anti-sheep IgG AF-647 (A21448, Invitrogen)

Fluorescence colocalization measurements

To provide additional evidence of thrombin production, EC surface membranes were stained with antibodies to co-detect FII/thrombin and thrombomodulin. The proteins were detected on EC surfaces (using primary antibodies and fluorescently labeled secondary antibody combinations listed above) and analyzed for colocalization to determine protein surface interactions. The entire merged image was analyzed for red and green signal similarities in shape, location, and intensity.

Preceding colocalization analysis, the cell images were processed for empirical background subtraction. Fluorescence cross-talk and bleed-through were minimized by

selecting fluorophores on secondary detection antibodies with non-overlapping spectra paired with narrow bandwidth filters, as previously described.[1]

The degree of similarity between detection in the red and green channels in merged images was determined using the fluorescence colocalization module within IP Lab software (Scanalytics, Inc.). The colocalization module calculates values for Pearson's correlation coefficient (PCC), that contributes shape information based on intensity distribution, and Manders' coefficients (M1 and M2), that describes the frequency that the red and green signals are detected in the same location throughout the merged image.[2-5] A thorough discussion of the mathematical theory has been reported by Comeau, et al.[3] Because PCC and M1 and M2 are determined in different mathematical ways, a positive calculated value (0.5 or higher) for either PCC or M1 and M2 indicates colocalization.[4,5] Intensity scatter plots were also used to visualize the extent of signal co-localization. A merged image with equal red and green intensities and perfect distribution will result in a scatter plot with a centered line with a PCC = 1.[4,5]

References

1 Turner, N. A. & Moake, J. L. Factor VIII Is Synthesized in Human Endothelial Cells, Packaged in Weibel-Palade Bodies and Secreted Bound to ULVWF Strings. *PloS one* **10**, e0140740, doi:10.1371/journal.pone.0140740 (2015).

2 Bolte, S. & Cordelieres, F. P. A guided tour into subcellular colocalization analysis in light microscopy. *Journal of microscopy* **224**, 213-232, doi:10.1111/j.1365-2818.2006.01706.x (2006).

3 Comeau, J. W., Costantino, S. & Wiseman, P. W. A guide to accurate fluorescence microscopy colocalization measurements. *Biophysical journal* **91**, 4611-4622, doi:10.1529/biophysj.106.089441 (2006).

4 Dunn, K. W., Kamocka, M. M. & McDonald, J. H. A practical guide to evaluating colocalization in biological microscopy. *American journal of physiology. Cell physiology* **300**, C723-742, doi:10.1152/ajpcell.00462.2010 (2011).

5 Zinchuk, V. & Grossenbacher-Zinchuk, O. Recent advances in quantitative colocalization analysis: focus on neuroscience. *Progress in histochemistry and cytochemistry* **44**, 125-172, doi:10.1016/j.proghi.2009.03.001 (2009).
